# Sexual dimorphism dominates divergent host plant use in stick insect trophic morphology

**DOI:** 10.1186/1471-2148-13-135

**Published:** 2013-07-03

**Authors:** Denis Roy, Ole Seehausen, Patrik Nosil

**Affiliations:** 1 Great Lakes Institute for Environmental Research, University of Windsor, Windsor, ON N9B 3P4, Canada; 2 EAWAG, Swiss Federal Institute of Aquatic Science & Technology, Department of Fish Ecology & Evolution, Centre for Ecology, Evolution & Biogeochemistry, Seestrasse 79, CH-6047, Kastanienbaum, Switzerland; 3 Division of Aquatic Ecology & Evolution, Institute of Ecology & Evolution, University of Bern, Baltzerstrasse 6, CH-3012, Bern, Switzerland; 4 Department of Animal and Plant Sciences, University of Sheffield, Sheffield S10 2TN, UK; 5 Institute for Advanced Study, Wissenschaftskolleg, Berlin, Germany

**Keywords:** Sexual dimorphism, *Timema cristinae*, Trophic morphology, Mandibles, Geometric morphometrics, Bayesian clustering, Morphological uniqueness, Occupied morphospace, Disruptive selection, Selection dissipation

## Abstract

**Background:**

Clear examples of ecological speciation exist, often involving divergence in trophic morphology. However, substantial variation also exists in how far the ecological speciation process proceeds, potentially linked to the number of ecological axes, traits, or genes subject to divergent selection. In addition, recent studies highlight how differentiation might occur between the sexes, rather than between populations. We examine variation in trophic morphology in two host-plant ecotypes of walking-stick insects (*Timema cristinae*), known to have diverged in morphological traits related to crypsis and predator avoidance, and to have reached an intermediate point in the ecological speciation process. Here we test how host plant use, sex, and rearing environment affect variation in trophic morphology in this species using traditional multivariate, novel kernel density based and Bayesian morphometric analyses.

**Results:**

Contrary to expectations, we find limited host-associated divergence in mandible shape. Instead, the main predictor of shape variation is sex, with secondary roles of population of origin and rearing environment.

**Conclusion:**

Our results show that trophic morphology does not strongly contribute to host-adapted ecotype divergence in *T. cristinae* and that traits can respond to complex selection regimes by diverging along different intraspecific lines, thereby impeding progress toward speciation.

## Background

Recent studies clearly demonstrate ecological causes for speciation [1-3], but extensive variance has also been noted in how far this process proceeds [4-6]. Speciation is often considered a continuum of divergence ranging from continuous variation among individuals within one species to complete discontinuity among groups in genetic, physiological and phenotypic adaptations to specific environments. The degree of divergence among taxa can be quantified along this continuum, typically by estimating for example; levels of reproductive isolation, genetic dissimilarities or the amount of lineage sorting among groups [5-7]. This continuum analogy can lead to the misconception that all diverging taxa, given enough time, will eventually complete ecological speciation. However, divergence in many systems can reach points whereby some level of divergence has been achieved but is balanced by other factors that prevent it from moving further [5-8].

Observed variation in the progress towards ecological speciation may thus be due to differential divergence time, degree of gene flow, strength of divergent selection, and the inherent genetic architectures underlying adaptive phenotypes [5-8]. However, another factor may be the number of ecological dimensions, traits or genes upon which divergent selection acts [3-6,8,9]. All else being equal, speciation may proceed further when divergent selection acts on a greater number of ecological axes, traits, or genes (multifarious selection, c.f. [4,5,9]). Selective pressures, however, can also act differently on different traits segregating them along different intraspecific factor (e.g., between sexes or between populations) or, alternatively, can lead to the expression of variable morphologies from a common genotype through the development of phenotypic plasticity [10,11]. Thus, divergence can occurs to varying degrees among various traits, with some contributing more toward population divergence and speciation than others [4,5,8,11,12].

Of the possible traits involved in ecological speciation, feeding morphology has been shown to play a role in a wide array of taxa [2,13-18]. Theoretical work suggests that under conditions where the most common phenotype depletes the most abundant resource, density-dependent disruptive selection on resource use traits can give rise to phenotypic variants adapted to the extremes of the resource spectrum [11,12,19,20]. This scenario favours extreme phenotypes that dissipate density-dependent selection regimes leading to resource partitioning among groups and, if these traits are also related to reproductive isolation, to resource based ecological speciation [11,12,19,20]. However, population divergence is not the only possible means of dissipating density-dependent selection. Density-dependent disruptive selection can also generate trophic traits that are sexually dimorphic, developmental stage specific, or even highly plastic [10-12]. In such cases the development of reproductive isolation based on trophic trait divergence is less likely and may even be hindered from developing based on other traits as well [11,12,20,21]. Theory also posits that occurrence of any one of the above alternatives will alleviate selection and thus limit the impetus for further divergence in the same trait(s) along any other factors (e.g., sex, ontogenetic stage, population) [11,12,19-21]. Beyond theory, however, empirical studies are needed to clarify along which factors disruptive selection drives trophic morphological divergence. In particular, studies conducted in systems known to be undergoing ecological speciation can test whether trophic divergence contributes to the speciation process [4-6,22].

To address these questions concerning trophic divergence and ecological speciation we quantify mandible shape variation (known trophic traits in many insects; [16,23]) in multiple populations of *T. cristinae* that exhibit two ‘ecotypes’ found living on two different host-plant species. These ecotypes exhibit adaptive genetically based divergence in colour, colour-pattern, body size and shape [24-26] and also partial reproductive isolation [27-29]. Thus, we might expect host-associated divergence in trophic morphology to follow suit, although this has not been examined in past work. Here, we assess the relative contribution of four explanatory factors on mandible shape variation: (1) host-plant use in nature, (2) host rearing environment in the laboratory, (3) amount of gene flow into populations from populations on the alternate host, and (4) sex. Our design allows identification of both genetic and environmental (phenotypic plasticity) effects on morphological variation. We assess shape variation in multi-dimensional morphological space (morphospace) using traditional and more novel shape-based analyses. Contrary to expectations, we find little host-associated divergence in mandible shape. Instead, the main predictor of mandible shape variation is sex, with lesser roles of genetic background, rearing environment and host use. Although our results suggest that divergence in traits along one intraspecific factor may limit trait divergence along other factors, they also challenge the notion that divergence along one factor completely inhibits that expressed in others [12,19,20]. Our results thus provide new insights into ecological speciation in this system, and on the role of sexual dimorphism in the adaptive divergence of trophic morphology in general.

### Study system: Ecological speciation in *Timema cristinae*

*Timema cristinae* is a species of wingless insect from the chaparral of southwestern North America whose individuals feed and mate exclusively on the host plants on which they live [30,31]. This species exhibits two ecotypes defined by the host plant species on which they are found (i.e., ‘*Ceanothus*’ and ‘*Adenostoma’* ecotypes; see [29]). Previous work has shown heritable morphological differences between ecotypes that have evolved in response to divergent selection for crypsis exerted by visual predators such as lizards and birds. In contrast to morphological adaptations for crypsis, divergent physiological adaptation to the different hosts is lacking, with both ecotypes exhibiting higher fecundity on the same host (*Ceanothus*) when predators are absent [29,32]. The ecotypes are in the process of ecological speciation, with pairs of populations on different host-plant species exhibiting stronger reproductive isolation than pairs on the same host [27,28,33]. Moreover the degree of both adaptive divergence and reproductive isolation is inversely related to levels of gene flow between ecotypes [24,25,34]. Given this evidence for host-mediated ecological speciation and for constraining effects of gene flow on divergence, we might expect both processes to also influence trophic morphology.

The two host plants used by *T. cristinae* offer very different properties in general structure, physiology and leaf characteristics [35]. *Ceanothus spinosus* (hereafter *C*) is a tree-like Rhamnaceae with thick broad leaves, high water conductance and relatively low mechanical strength. *Adenostoma fasciculatum* (hereafter *A*) meanwhile, is a bush-like Rosacea, characterized by thin tough needle-like leaves with low water conductance but high mechanical strength [35]. Given these differences in food resources, *T. cristinae* populations adapted to the different hosts might possess specific feeding morphologies adapted to these differences. This is especially likely considering past work demonstrating mandible shapes in other insects to be related to the toughness and hardness of their diets [23,36,37]. If this were the case in *T. cristinae*, it would demonstrate at least two different ecological dimensions (trophic ecology and predator avoidance) working in the same direction during ecological speciation. Alternatively, if trophic divergence is manifest primarily along non-host associated lines, then it will have diverged along a different trajectory relative to traits involved in crypsis and may stymie how far ecological speciation proceeds.

## Methods

### Sample collection & preparation

**Figure 1:**
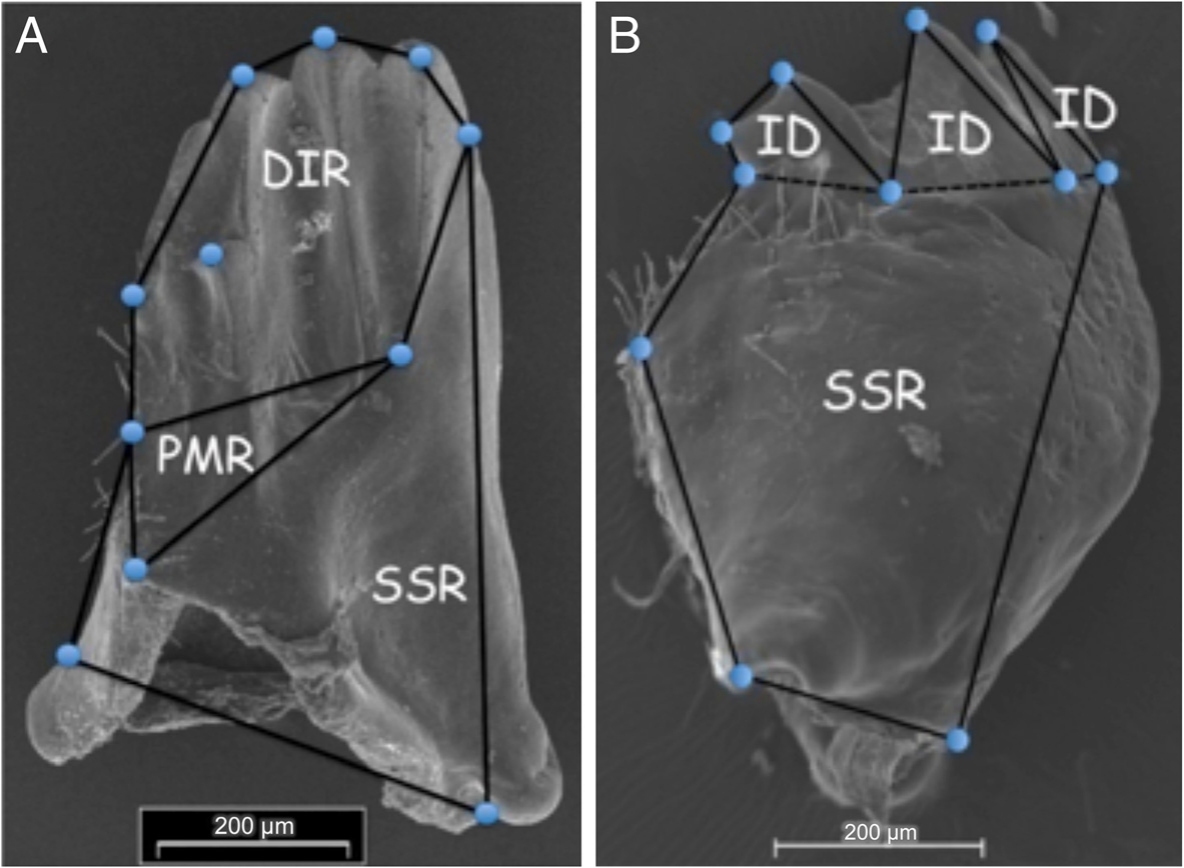
**Electron micrographs of *Timema cristinae *mandibles showing features outlined by landmarks used. (A)** Lingual view showing distal incisor region (DIR), the proximal molar region (PMR) and the structural support region (SSR). **(B)** Occlusial view showing the incisor dents and the SSR. Consensus configuration of all specimen corrected landmark configurations of the lingual and occlusial views are superimposed on mandible figures.

A total of 200 sexually mature individuals were randomly chosen from previously collected and preserved samples of six populations (*N≅*25-50/population, Table 1 for details) that are the focus of past and ongoing research [25,38], and which represent differential host use and levels of geographic isolation. Sexes can be unambiguously differentiated in mature individuals by stark differences in external genitalia [30,39]. A subset of individuals from each population (range = 3-25) were reared from first-instar to sexual maturity on either their native and/or alternate hosts under laboratory conditions, as described in previous work [25,38]. Left and right mandibles were excised from specimens under a dissecting microscope, cleaned by bleach immersion and mounted onto scanning electron microscope (SEM) stubs in a specific orientation (Figure 1). Each individual’s mandible orientation was calibrated against an initial set, which we used as our experimental standard. Micro-adjustments to the placement of mandibles on the stub or to the ESEM stand were used to achieve standard orientation. Broken mandibles or ones whose final orientation was too different from the standard were removed from the analyses. To acquire better overall coverage of total shape, left and right mandibles were set in different perspectives accentuating different features. Left mandibles were placed in a lingual view emphasizing more functional features: distal incisor region (DIR), the proximal molar region (PMR) and structural support region (SSR; Figure 1A). Right mandibles were placed in an occlusial view focussing on the SSR but also showing the incisor dents (ID; [36]; Figure 1B). SEM images were generated using a Philips® FEI ESEM scanning electron microscope set at 60-70KV and a scale factor of 200 μm. Table 1 summarizes the final count of individuals used in each population under various rearing conditions.

**Table 1:** **Summary of *Timema cristinae *used in this study**

**Population**	***N *total**	**Host**	**FLD**		**LAB-*A***		**LAB-*C***		*Influence
			**M**	**F**	**M**	**F**	**M**	**F**	
*Lingual*									
HVA	24	A	17	2	-	-	5	-	15.97
LA	45	A	1	3	14	8	11	8	0.21
LRN	18	A	11	4	-	-	-	3	0.39
OUTA	24	A	16	2	-	-	5	1	119.89
PRC	26	C	15	1	-	-	10	-	2.62
VPC	52	C	10	2	14	11	12	3	19.46
*Occlusial*									
HVA	23	A	17	2	-	-	4	-	15.97
LA	46	A	2	3	13	8	12	8	0.21
LRN	17	A	9	5	-	-	-	3	0.39
OUTA	23	A	15	2	-	-	5	1	119.89
PRC	26	C	15	1	-	-	10	-	2.62
VPC	49	C	10	1	14	11	10	3	19.46

Host: *A-Adenostoma fasciculatum, C-Ceanothus spinosus*; Rearing conditions: FLD = Field, LAB = Laboratory; *- Calculations of influence are described in the text and equal a proxy for gene flow, with larger values reflecting more gene flow into a population from the alternate host.

### Geometric morphometrics

Electron micrographs were subjected to geometric morphometric analyses using 11 homologous landmarks chosen to outline the features listed above. Left (hereafter lingual) and right (hereafter occlusial) mandibles were analysed separately. Standardized landmark configurations were imported into MorphoJ [40], where a generalized least squares Procrustes superimposition (GPS) was performed. The GPS generated a consensus configuration for both mandibles and a thin-plate spline was used to quantify deviation of individual specimen landmark configurations from the consensus along principal axes of shape change [40,41]. Deviations from the consensus along principal axes generate partial warps quantifying individual based shape changes [41,42]. For both mandibles, the thin-plate spline produced 16 partial warps and two uniform components per individual, representing local and overall shape variables, respectively. All 18 shape variables were used in subsequent analyses.

### Shape variable size correction

Centroid sizes of the lingual mandibles were transformed to their natural logarithm to fit a normal distribution while no such transformation was required for the occlusial. Centroid sizes were tested for differences among four explanatory factors: sex, host-adapted ecotype, population of origin, and rearing condition. In both cases, females exhibited significantly larger mandibles than males (lingual; females = 992.67±64.84μm vs. males = 876.75 ± 50.83μm; *t*_68.66_ = −11.13, *P* < 0.001: occlusial; females = 953.35 ± 67.83μm vs. males = 850.87 ± 44.76μm; *t*_59.91_ = −9.56, *P* < 0.001). No significant differences were found in centroid sizes with respect to other tested factors. Because size is known to influence shape through allometric trajectories [41,43,44], shape variables were regressed against centroid size using MANCOVA approach and residuals were used in subsequent analyses [40,44,45]. Multivariate size correction used a single regression for both sexes because the interaction term between sex and size (used as covariate) did not account for significant shape variation (*P* > 0.05).

### Bayesian shape analyses and morphological clustering

Using all data for each mandible orientation (lingual *N* = 189; occlusial *N* = 184), we assessed the most likely number of statistically different groups within samples based solely on their shapes using a Bayesian posterior probability assignment tests run in the program Autoclass-C v 3.3.4. This analysis allowed us to statistically find the most obvious separation in the data without prior grouping by assessed factors. Detailed descriptions of Autoclass-C are available from the NASA home page (http://ti.arc.nasa.gov/tech/rse/synthesis-projects-applications/autoclass/autoclass-c/) and in Cheeseman & Stutz (1996). Briefly, Autoclass-C uses a Bayesian extension of finite-mixture modelling to perform unsupervised searches recovering the most probable number of statistically different groups given the data. Searches make no prior assumptions of actual group number but assume each resulting group fits a given distribution set by the user [46]. In each iteration, Autoclass-C generates a number of hypothetical distributions with given parameters (e.g., means and variances) to which actual data are permutated and fit over a given number of cycles. Convergence is achieved in each iteration when the actual data fit the hypothesized distributions within a given error estimate over a predefined number of cycles. The probability of the converged data is then evaluated using a Bayesian framework. We used 10 000 000 iterations allowing each to reach convergence over 100 000 cycles where convergence was deemed acceptable when actual and hypothesized parameters were within 0.0025 over at least 10 consecutive cycles. Searches recorded the most probable number of groups in the data every 10 000 iterations and saved the best 200 overall. We assumed variables used in the modelling fit normal or lognormal distributions and that variable-specific error terms were fixed. Variable-specific error terms were calculated using all individuals included in the analyses. Autoclass-C was initially run using all shape variables but was also run using the same settings on relative warps (RWs) (PCs of unweighted shape variables; [41]). Results from both analyses were identical and so those using RWs, which fit model assumptions better, are presented. Autoclass-C results also generated individual-based posterior probabilities of belonging to recovered groups which were then used to generate probability of assignment plots [47]. Mean RW scores of individuals belonging to dominant clusters were also used to generate deformation grids outlining group specific shapes.

### Uniqueness of occupied morphospace

Multivariate parametric analyses are most reliable when sample sizes among and within grouping factors are well balanced [41,48]. Such balanced designs, however, can be difficult to achieve for small complex morphological features (~ 620 μm total length), which are delicate, costly to prepare and not easily replaced. Because our overall data set was not conducive to parametric assessments of variance partitioning, we chose to compare shape differences within explanatory factors by assessing morphological uniqueness. Morphological uniqueness (hereafter MU) quantifies the amount of unique morphological shape space occupied by two predefined groups. MU is based on the non-parametric niche overlap index developed to estimate the overlap between groups based on quantitative functional traits [49]. Briefly, along each RW, each individual’s score is converted to a kernel distribution which contributes to an overall kernel density function formulated for the group to which it belongs [49]. Group specific functions for each RW are then compared by stepwise integration of the intersecting area between the two functions over the predefined range given by the maximum range of the largest group. This integral determines the overlap between the two groups along this particular RW [49,50]. Because the functions are bounded over the same range, the uniqueness along a RW can be considered unity minus the overlap. The uniqueness calculated over each RW is then weighted by the amount of variance accounted for by each RW (determined from eigenvalues) and summed. Generated MU indices range between 0 and 1 quantifying non-overlapping morphospace occupied by the two compared groups. Significance of MUs between groups is assessed by resampling the data for the same number of individuals but arbitrarily assigned to the different groups (with replacement) one thousand times. Because the MU can be applied to the same data but testing different explanatory factors, it can quantify how each explanatory factor partitions the same morphospace defined by a given variable (here RWs) or set of variables outlining more general morphospace. Here, overall MUs were calculated and compared among all groups within an explanatory factor (i.e., sex, host, population, and rearing condition) in a pairwise fashion and permutations were performed using R scripts (available from the authors; [51]). MUs for each explanatory factor were also calculated for each RW to assess which factor best partitioned the variance along each. In analyses including factors with more than two groups (i.e., population and rearing condition), the mean MUs from all pairwise comparisons along each RW were used in comparisons with other factors (see Table 2). Deformation of the consensus configuration along the first three RWs was also determined by regressing individual shape variables onto RW1, RW2 and RW3 scores, respectively [40,52,53].

**Table 2:** **Decomposed raw Morphological Uniqueness of *T. cristinae *mandibles quantified among all tested factors**

**RW**	**% σ**	**Sex**	**Host**	**Population**	**Rearing**	**Dominant factor**	**% σ**	**Sex**	**Host**	**Population**	**Rearing**	**Dominant**
					**condition**						**condition**	**factor**
	*Lingual*						*Occlusial*					
1	31.38	0.087	0.164	0.299	0.201	P	27.81	0.514	0.177	0.310	0.231	S
2	14.62	0.511	0.149	0.255	0.301	S	14.25	0.112	0.105	0.290	0.216	P
3	11.02	0.156	0.300	0.259	0.218	H	13.90	0.180	0.192	0.196	0.164	P
4	8.92	0.565	0.134	0.231	0.200	S	8.19	0.410	0.069	0.274	0.140	S
5	7.60	0.195	0.098	0.218	0.140	P	6.86	0.124	0.088	0.151	0.212	R
6	6.04	0.757	0.180	0.274	0.232	S	6.05	0.167	0.121	0.240	0.164	P
7	4.91	0.463	0.127	0.239	0.143	S	4.72	0.522	0.066	0.283	0.250	S
8	4.19	0.076	0.114	0.151	0.179	R	4.28	0.491	0.144	0.194	0.170	S
9	3.13	0.675	0.150	0.226	0.112	S	3.21	0.150	0.120	0.217	0.101	P
10	2.01	0.503	0.148	0.250	0.201	S	2.86	0.519	0.109	0.173	0.136	S
11	1.42	0.383	0.082	0.196	0.129	S	2.33	0.212	0.074	0.165	0.217	R
12	1.23	0.117	0.113	0.196	0.195	P	1.47	0.677	0.118	0.195	0.191	S
13	1.00	0.130	0.106	0.215	0.120	P	1.08	0.672	0.148	0.238	0.227	S
14	0.71	0.226	0.074	0.216	0.183	S	0.86	0.362	0.065	0.193	0.149	S
15	0.64	0.374	0.175	0.254	0.201	S	0.67	0.380	0.115	0.218	0.129	S
16	0.47	0.203	0.210	0.206	0.167	H	0.61	0.171	0.109	0.216	0.167	P
17	0.39	0.136	0.071	0.182	0.166	P	0.50	0.142	0.078	0.189	0.144	P
18	0.30	0.129	0.125	0.199	0.170	R	0.33	0.315	0.153	0.256	0.110	S
RW_DOM_		9	2	5	2			10	0	6	2	

Row entries describe divergence along each Relative Warp (PC of shape variables) determined from pairwise comparisons within each tested factor. Population and rearing condition entries show means calculated over 15 and 6 comparisons, respectively. Calculated using all individuals (*N* = 189 lingual and *N* = 184 occlusial). H = host, P = population, R = Rearing condition, RW = Relative warp, RW_DOM_ = number of dominant relative warps (does not consider the variance explained by specific RWs), S = sex,% σ = percent variance explained along RW.

### Genetic and environmental basis of mandible shape

To quantify the relative importance of genetic background (e.g., population of origin) and rearing environment on mandible shape(s), we used individuals from populations LA and VPC reared from first instar to sexual maturity on both hosts in a reciprocal transplant experiment performed in the laboratory (Table 1; [25]). Landmark configurations for these samples were isolated and used to generate a new consensus configuration and a new set of size corrected shape variable as described above. We then used MANOVA to determine the influence of sex, population of origin, host rearing condition and their interactions on mandible shape variation. The population effect tested influences of genetic background while host rearing condition tested environmental effects. Interaction terms tested sex specific responses in the different populations and hosts, and population specific responses to hosts [25,54]. MANOVA was performed using default settings and a type III sum of squares model taking into account uneven sample sizes in STATISTICA 64 v.10 (Stat Soft Inc., Tulsa, OK USA).

### Sex removal

Results of the analyses revealed an overwhelmingly strong effect of sex on mandible shape (see results). However, other factors also explained some portion of mandible shape variation. To justify the consideration of a single sex (male) in subsequent analyses and to relate findings to the entire population, we compared the shape changes occurring in all males and females in both common and global morphospace using ordered axes analysis [55] and the assessment of angular differences along RWs between sexes [41,55]. Results from both analyses indicated that males and females, although differing in overall shape, followed the same shape change trajectories through both common and global morphospace (additional file 1). Consequently, data were sub-divided by sex and Bayesian assignments, MU and MANOVA tests described above were performed using only field-collected (wild) males from populations with nine or more individuals. In this case, MANOVAs nested populations within host as specific populations use only a single host in the wild.

### Gene flow

Finally, we tested the influence of gene flow between hosts-adapted populations on mandible shape variation. Isolated populations with low levels of gene flow might be expected to exhibit less shape variation than those characterized by higher gene flow. Using only wild males from populations with nine or more individuals (Table 1), we generated another consensus configuration and set of shape variables. Shape variables were converted to RWs as above and population-specific standard error of the mean of RW1-RW3 scores (an index of shape dispersion) were regressed against a population-specific index of influence, a proxy for gene flow. The index of influence for each population was calculated as the sum of the proportion of the area of the focal population and its adjacent population occupied by the alternate host (determined from aerial photographs; [56]) divided by the sum of the distance between them. Previous work has demonstrated that the proportion of aerial coverage occupied by the alternate host is highly correlated with levels of gene flow inferred from molecular data [25,28,56,57].

## Results

### Bayesian assignment by shape

**Figure 2:**
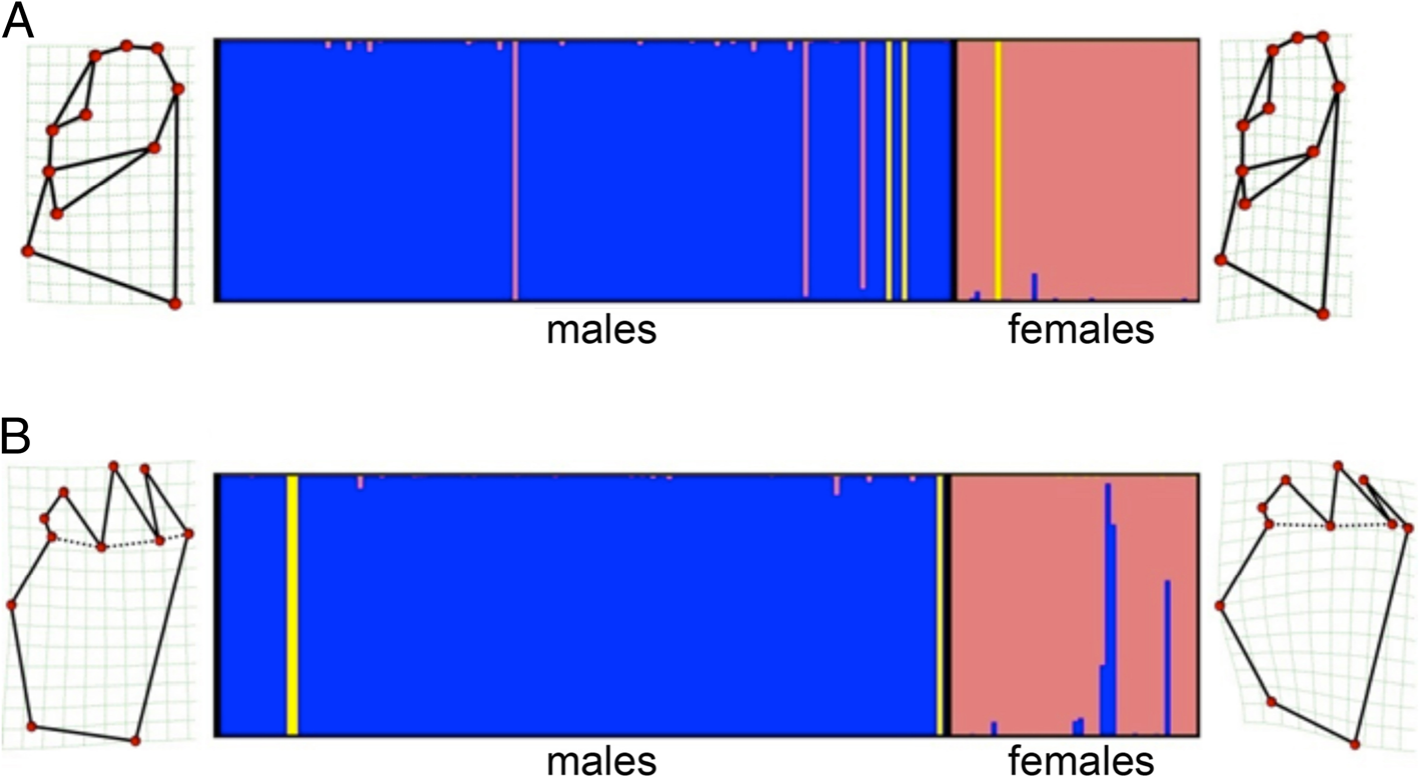
**Bayesian posterior probability plots estimating number of clusters among sampled individuals based on lingual (A) and occlusial (B) mandibles shapes.** Each individual is represented by a bar whose colouration is based on its probability of belonging to each cluster. Deformation grids associated with the two main clusters derived from the average shape of individuals belonging to each.

From the overall dataset of both mandibles, the most likely number of independent groups recovered from unsupervised Bayesian searches separated individuals into three clusters with two dominating the assignments and one encompassing only three individuals (Figure 2). In both cases, the two dominant clusters were strongly associated with sex indicating that sexes were the most obvious separation in the data. The third minor cluster was likely artifactual resulting from Autoclass-C’s sensitivity in generating groups with such small fixed error terms, as its membership was not consistent between mandibles. In lingual mandibles, 97% of males were assigned to one cluster with greater than 95% posterior probability, while 96% of females were assigned to a different cluster also with greater than 95% posterior probability (Figure 2A). The best classification was 2.64 times more probable than the next best classification scheme separating individuals into the same clusters and 104.27 times more probable than the classification estimating the next most probable number of clusters (two; male and female only). Assignments for occlusial mandibles were similar to those of the lingual, but varied in cluster statistics (Figure 2B). Ninety-one percent of females were assigned to the same cluster with greater than 95% posterior probability. Ninety-eight percent of males were assigned to a single cluster with greater than 95% posterior probability. The final best classification was 163.04 times better than the next most probable classification and 160 000 times better than the classification estimating the next most probable number of clusters (two; male and female only). Lingual deformation grids showed that for the same overall sized mandible, the female’s was generally narrower with a smaller proximal molar region (PMR), structural support region (SSR) and more pointed distal incisor region (DIR) (Figure 2A). Average male occlusial mandibles appeared to have more streamlined SSR and longer more extended incisor dents (ID) (Figure 2B).

### Factor-specific MU on overall data

**Figure 3:**
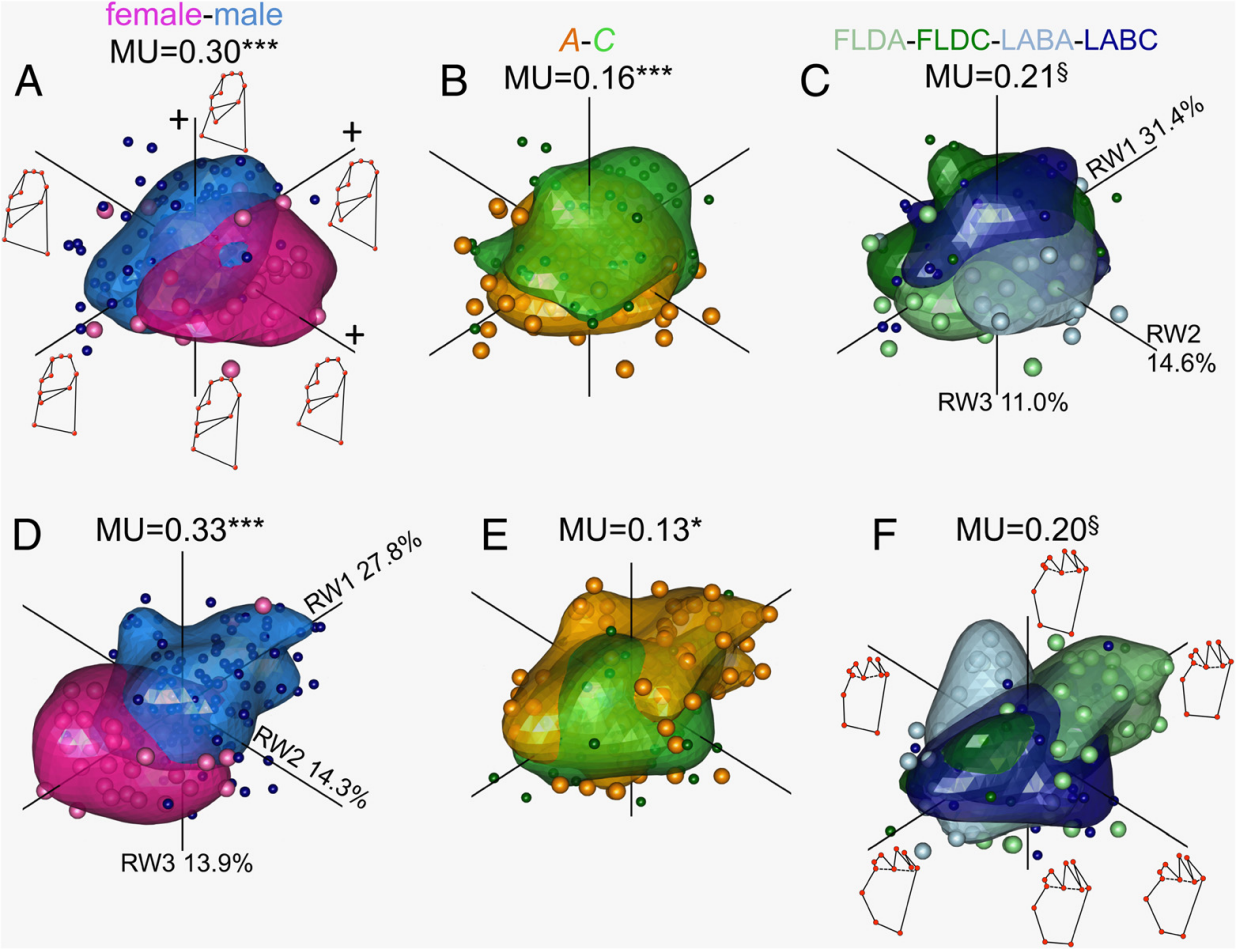
**Morphological uniqueness (MU) assessed for *T. cristinae *lingual and occlusial mandibles using all 18 relative warps (RWs) supported by 1000 resampling permutations.** Panels show 80% confidence bubbles outlining morphospace occupied by different **A)** sexes **B)** host plant ecotypes and **C)** rearing conditions for the lingual mandible along the first three RWs accounting for 57% of overall shape variation. Panels **D-F** show the same as **A-B** but for the occlusial mandible whose first 3 RWs account for 56% of the shape variation. Deformation grids next to RWs in panel **A** and **F** show general shape change trends along each. FLD = field LAB = Laboratory *A* = *Adenostoma* and *C* = *Ceanothus*; §-Mean MU calculated from all pairwise comparisons; *** = *P* < 0.001, ** = *P* < 0.01, and * = *P* < 0.05.

In both mandibles, sex best partitioned the overall morphospace assessed along all 18 RWs (overall MU), followed by mean pairwise differences between populations and rearing conditions, while host-adapted ecotypes partitioned the morphospace the least (Figures 3, Additional file 1: S2 and S3). Although sex best partitioned the morphospace along a majority of individual RWs (RW-specific MU), other factors also partitioned morphospace along the same RWs, but to a lesser degree (Table 2). This result supports the notion that all factors interact to some extent but that one typically dominate the divergence (Table 2). After sex, mean pairwise population comparisons best partitioned morphospace along a larger number of RWs followed by mean pairwise rearing condition comparisons and then host-adapted ecotypes (Table 2). In the morphospace outlined by the first three RWs, sexes were clearly most separated along RW2 for the lingual mandible but along RW1 for the occlusial. In both cases shape differences along these RWs, as depicted from deformation grids along RWs, followed similar trends as that determined in the Bayesian clustering (compare Figures 2 and 3). When populations and rearing conditions occupied significantly different morphospace, separation was typically most obvious along RW1 and RW2. However, many populations also showed substantial overlap consistent with non-significant MUs (e.g., Figure 3C,F and Additional file 1: Figures S2, S3). In both mandibles, populations sharing the same host tended to have larger MUs than those on alternate ones (MU_*same*_ = 27, MU_*different*_ = 24.75). Similarly, the largest observed differences in MU among rearing conditions occurred between those both using *A* as host (i.e., lab versus field environments), while that quantified on *C* were not significant. Thus, in both population and rearing condition comparisons, anticipated higher MUs on alternate hosts was not observed, indicating a low level of host influence in these factors. Despite their significant MUs, no obvious separation was observed along any of the three RWs when considering host-adapted ecotypes (Figure 3B,E), indicating limited host-specific shape adaptation when all data are considered together.

### Genetic and environmental basis of mandible shape

MANOVA results from the reciprocal transplant experiment revealed a pattern similar to the overall dataset. Namely, sex accounted for most of the shape variation for both mandible orientations (Table 3). However, significant population (genetic) and host rearing condition (environmental) components were also detected. For the lingual mandible, genetic effects accounted for a greater portion of the variance than did environmental ones, while for the occlusial, both factors accounted for similar variance proportions. Both mandibles also demonstrated a significant interaction in shape variation between population and host implying that one population was more responsive to environmental differences than the other (Table 3). No sex specific influences of population or host rearing condition were observed in either mandibles indicating that both sexes responded similarly to genetic and environmental differences. The power of these interactions to reject the null, although low, was nevertheless above 35% in all cases [48], and results are consistent with those of the ordered axes analyses showing similar shape change trajectories in both sexes regardless of other factors in the overall data (see Additional file 1: Figure S1).

**Table 3:** **MANOVA testing the genetic and environmental basis of mandible shape in *Timema cristinae *(using LA and VPC reared under laboratory conditions)**

**Sources of variation**	**Wilk’s**	***F***	**df**		***P***	**Partial variance explained**	**Observed power**
	**Λ**		**H**_**o**_**, Error**				
						**(%, η**^**2**^**)**	
*Lingual*							
Sex	0.19	13.15	18	57	<0.001	80.6	1.00
Population (Popn)	0.25	9.30	18	57	<0.001	74.6	1.00
Host rearing condition (HRC)	0.54	2.65	18	57	0.003	45.6	0.99
Sex x Popn	0.65	1.68	18	57	0.072	-	0.87
Sex x HRC	0.82	0.70	18	57	0.794	-	0.42
Popn x HRC	0.49	3.26	18	57	<0.001	50.7	0.99
*Occlusial*							
Sex	0.28	7.69	18	55	<0.001	71.6	1.00
Popn	0.36	5.49	18	55	<0.001	64.3	1.00
HRC	0.35	5.68	18	55	<0.001	65.0	1.00
Sex x Popn	0.78	0.85	18	55	0.682	-	0.36
Sex x HRC	0.84	0.60	18	55	0.855	-	0.52
Popn x HRC	0.44	3.83	18	55	<0.001	55.6	1.00

*η*^*2*^- values indicate the amount of variance attributable to tested factors;

*F* - approximation to F-ration derived from reported Wilk’s Λ and degrees of freedom.

### Host- and population-specific divergence within sex

**Figure 4:**
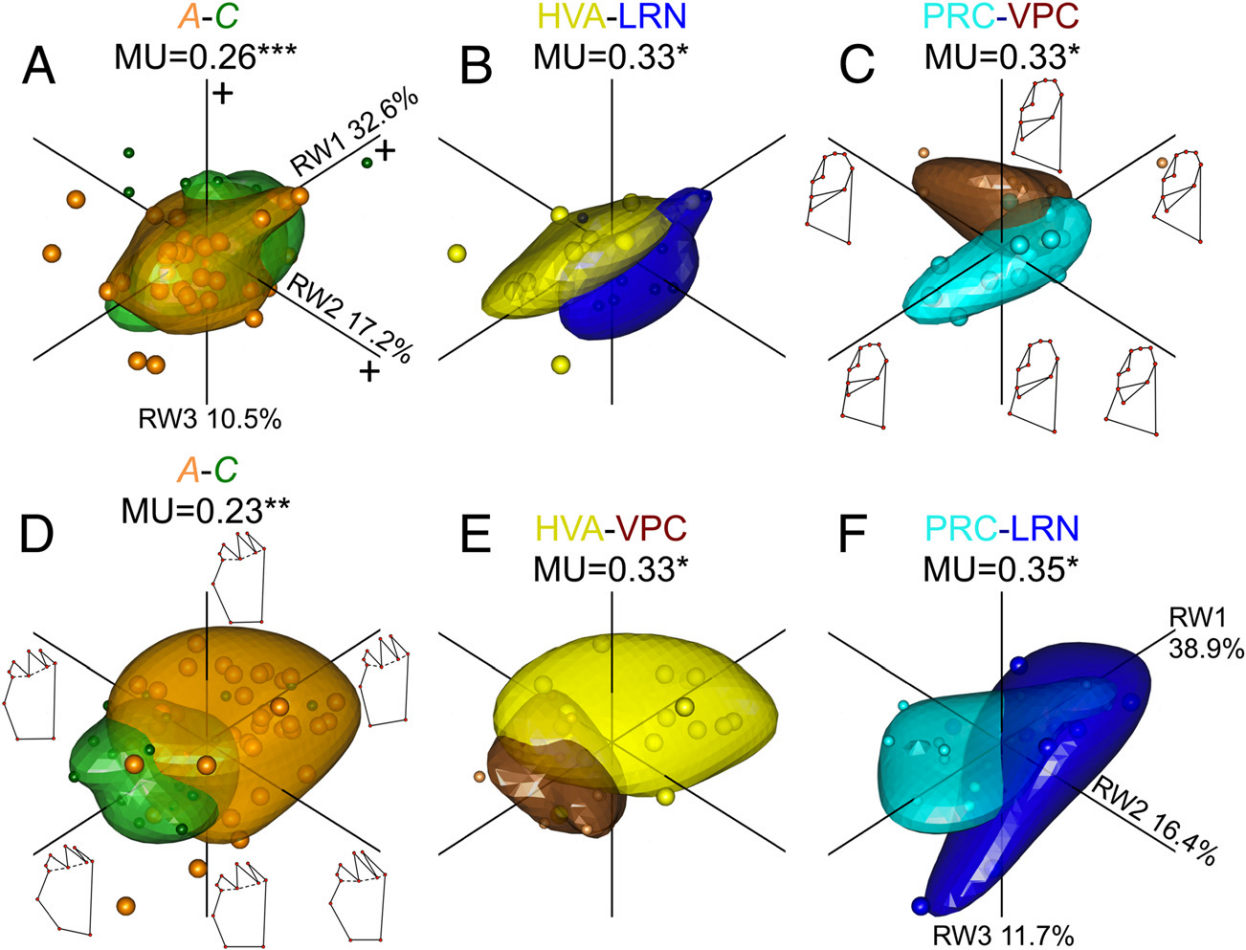
**MU of field reared male *T. cristinae *lingual and occlusial mandible shapes assessed using all 18 RWs supported by 1000 resampling permutations.** Panels show 80% confidence bubbles outlining morphospace occupied by different **A)** host plant ecotypes, **B** and **C)** key pairwise populations comparisons along the first three RWs accounting for 60.3% of shape variation. Panels **D-F** show the same as **A-C**, but for the occlusial mandible whose first 3 RWs account for 67% of the shape variation. Deformation grids next to RWs in panel **C** and **D** show general shape change trends along each. *** = *P* < 0.001, ** = *P*< 0.01, and *=*P* <0.05.

Bayesian classification schemes of field-collected (wild) males based on shape variables for both mandibles recovered only a single most probable cluster. For the lingual and the occlusial mandibles, best classifications were 8 170 000 and 10.15 times more likely than the next best classifications predicting two cluster, respectively. Thus, Bayesian analyses could not further cluster the data when only a single sex was considered. In contrast to Bayesian results, MANOVA and MU analyses showed variable effects of host and population on mandible shapes. For the lingual mandible, MANOVA showed that host accounted for a larger portion of the overall variance than did populations nested within host (Table 4). For the occlusial mandible, though, host was not a significant explanatory factor, but population within host was (Table 4). MU estimated along all 18 RW did not agree with MANOVA results but rather showed that both host and population significantly partitioned outlined morphospace for both mandibles. In both cases, however, the MU calculated between host-adapted ecotypes was less than that expressed between individuals belonging to different populations, especially when only significant population comparisons were considered (Figure 4 and Additional file 1: Figures S4-S5). For the lingual mandible, although the MU was significant using all 18 RWs, no clear separation between host-adapted ecotypes was obvious in the morphospace outlined by the first three RWs, indicating a lack of host-specific shape as defined by the deformation grids along RW1-3 (Figure 4A). Population level MUs, meanwhile, were stronger and more obvious along RW2 where some populations had more pointed DIRs, more curved PMRs and broader SRRs than others (Figure 4B-C and Additional file 1: Figure S4). Half the significant population MUs recovered were between populations sharing the same host indicating that not all population differences were host-based. For the occlusial mandible, clear differences were observed along RW1 between host-adapted ecotypes where *A*-ecotypes scored more positively and exhibited more slender SSRs and longer IDs relative to *C*-ecotypes (Figure 4D). Populations exhibiting significant MUs were also most obviously different along RW1, where populations using *A* also scored more positively and shared the same features as *A*-ecotypes (Figure 4E-F and Additional file 1: Figure S5). All significant population MUs in the occlusial mandible were between those using alternate hosts suggesting a strong host-population interaction (Figure 4E-F and Additional file 1: Figure S5).

**Table 4:** **MANOVA testing mandibles shape variables among field collected male *T. cristinae***

**Sources of variation**	**Wilk’s**	***F***	**df**		***P***	**Partial variance explained (%, η**^**2**^**)**	**Observed**
	**Λ**		**H**_**o**_**, Error**				**power**
*Lingual*							
Host	0.34	4.96	18	47	<0.001	65.5	0.99
Population (Host)	0.18	2.02	54	140.86	<0.001	44.4	0.99
*Occlusial*							
Host	0.63	1.38	18	43	0.189	-	0.76
Population (Host)	0.18	1.90	54	128.94	0.002	44.1	0.99

*η*^*2*^- values indicate the amount of variance attributable to tested factors;

*F* - approximation to F-ration derived from reported Wilk’s Λ and degrees of freedom.

In summary, single sex analyses revealed variable levels of population and host mandible shape differences depending on the test performed (Bayesian, MANOVA or MU) likely reflecting more subtle shape differences than those observed between sexes. Pairwise population MUs were generally greater than those between host-adapted ecotypes, but host-population interactions, especially in the occlusial mandible, were recovered. Thus, host effects on mandible shape were slight and subtle at best.

### Gene flow

Mandible shape dispersion estimated from wild males was not significantly related to the potential for gene flow among neighbouring populations for either mandible orientation (*P* > 0.05; Additional file 1: Figure S6).

## Discussion

Here, we showed mandible shape differentiation between sexes and between host-adapted ecotypes, some of which was population specific. More slender occlusial mandibles with longer more extended cutting regions were associated with individuals using *Adenostoma* as host, whose resources are likely more difficult to access [35]. However, we also showed that the strongest predictor of mandible shape variation in this species is sex rather than host-adapted ecotype, population of origin or rearing condition. This sexual dimorphism was prevalent both in the overall data and in laboratory-reared (reciprocal transplant) individuals from both hosts, indicating its heritable nature. The degree to which genetics and the environment influenced mandible shape was variable, and depended on the mandible features emphasized in the two mandible orientations (lingual vs. occlusial). Once sexual dimorphism and rearing conditions were accounted for (wild male data), we showed that host played a significant yet variable role in determining mandible shapes, as did population of origin. Finally, and in contrast to most other morphological features measured in this species to date, no effect of gene flow was observed on mandible shape variation. Below, we further develop these issues and discuss their evolutionary and ecological implications.

### Potential causes of sexual dimorphism in *T. cristinae* mandibles

Sexual dimorphism is common among various animal taxa and is often attributable to divergent sexual and/or ecological roles between the sexes [58-61]. The causes of sexual dimorphism in trophic morphology in *T. cristinae* remain unknown but could be attributable to various mechanisms, including fecundity-based sexual size dimorphism, sexual selection, or differential feeding ecology between sexes.

### Sexual size dimorphism

Sexual size dimorphism can be a consequence of adaptation to different male and female reproductive roles [58-60]. These roles are often the result of fecundity selection on egg development, laying and nutrient allocation amongst others. For instance, female *Timema*, unlike males, ingest soil from below their host plant, which they use to coat egg cases. Consequently, the egg laying sex often grows faster and larger to compensate for the extra allocation of resources [59,60]. Because such traits are costly to express in the absence of fecundity selection, such fecundity-based adaptation can translate into size differences between sexes and into dimorphism based solely on allometry [59,60]. Previous work has demonstrated sexual dimorphism in other morphological traits of *T. cristinae* in relation to size, with female body size being larger than males [25,39]. Here, we found female mandibles to be significantly larger than those of males, consistent with sexual size dimorphism. However, strong sexual dimorphism was still present in the residuals of shape variable regression with centroid size, a procedure that should account for allometric differences between sexes [40,44]. Thus, the sexual dimorphism observed here goes beyond simple allometric differences. Moreover, in most reported instances of fecundity-based sexual size dimorphism, sexually dimorphic traits consistently co-vary with size differences (i.e., the larger sex also exhibits larger traits) [58,62]. Instead, we found that size-corrected mandibles showed much broader (lingual) and more extended (occlusial) features in males than in females, opposite to the overall dimorphic body size-trait covariance pattern. Thus, other factors than size are involved in the sexually dimorphic mandible shapes in this species.

### Sexual selection

Sexually dimorphic mandibles may be the result of sexual selection in efforts to sequester mating opportunities from conspecific rivals [59,63,64]. Within insect taxa in particular, male fighting behaviours can involve the use of mandibles [63,65]. Reports of male fighting in Phasmatodea (including *T. cristinae*) however, are rare but can arise during mate guarding where a male encounters an already coupled pair [66,67]. In these encounters, coupled males grip the female abdomen with their genitalia curving it away from approaching rivals to prevent copulation. Males may also engage in a ‘boxing’ behaviour using their front legs [67], but the use of mandibles in such interactions has not been reported. In addition, more recent *T. cristinae* mating behaviour work does not show any evidence of mandible use in mate pairing, courtship display, initiation of copulation, or post-copulatory mate guarding [68]. Thus, although the usefulness of mandibles as sexual weapons or traits conferring reproductive success in *T. cristinae* has not been explicitly tested, sexually dimorphic mandibles do not appear to be related to sexual selection in any obvious way.

### Ecologically based sexual dimorphism

Another explanation for sexually dimorphic mandibles is the development of sex-specific feeding ecologies, as reported in other species [69-72]. Although *T. cristinae* males and females carry out all life history stages on the same plant species, it may be that they partition food resources by feeding on different parts of the same host-plant. Such tissue specific feeding behaviours have been demonstrated among other plant-browsing insects [16,73], but not typically between sexes within the same species. We showed that female lingual mandibles were more slender and pointed relative to those of males. Studies have demonstrated that sharper more pointed mandibles are generally better at initiating and propagating fractures in tough leaf material [16,73]. Thus, female mandibles may be better adapted to feeding on the tougher parts of host plants whereas male mandibles may be better on the softer mesenchyme leaf tissue. Such sex-specific feeding strategies are also consistent with observed differences in occlusial mandibles wherein females had broader structural features with more curved cutting regions than males. Females may house larger mandibular muscles in enlarged structural regions capable of applying greater pressure to sharper cutting features enabling easier plant tissue fracture. Sex-specific feeding habits have not been tested in *T. cristinae*, but offer clear avenues for further research. Directed studies quantifying sex-specific diets using either dietary tracer information (i.e., stable isotopes and/or fatty acids) or tissues enriched with other chemical tracers could help determine whether mandible sexual dimorphism is diet based [74,75].

### Sex versus host and other factors

The large sexual dimorphism in *T. cristinae* mandible shape suggests that it may limit mandible divergence along other intraspecific factors such as host or population. Theoretical modelling suggests that the mechanism by which adaptive ecologically-based sexual dimorphism emerges may be the same as that driving adaptive ecological speciation or phenotypic plasticity [10-12,19,20]. In this context, the development of ecologically-based dimorphic sexes, ecologically divergent species, or ecology-based adaptive phenotypic plasticity, is thought to supplant the evolution of other alternatives by dissipating disruptive selection among traits [11,12,19,20]. The development of any one of these types of divergence should thereby alleviate the impetus for other types of divergence in the same trait(s) [10-12]. If mandible shape differences between males and females result from adaptation to different diets, then the mandible shape adaptations between sexes might limit further host-based (or other factor based) mandible shape adaptation (but see [12]). The large sexual divergence in mandible shapes observed in both the overall data and in the reciprocal transplant experiment attests to the dominance of sex in determining mandible shape. However, the lack of sex-specific effects of host rearing condition or population in the reciprocal transplant experiment indicate that genetic and environmental factors tend to influence both sexes similarly (i.e., that *T. cristinae* do not exhibit sex-specific adaptations to the different host plants and that the sexes do not feed on different hosts). This is further supported by the fact that although males and females accumulate variance in mandible morphology at different evolutionary rates, they do so along the same shape change trajectories in both common and global morphospace (see Additional file 1). Thus, mandible shape differences between sexes are not host plant-specific. Whether the sexes feed on different parts of the same host plant, however, is unclear (see above). Moreover, and although sex best partitioned the variation in mandible shape, it did not totally preclude mandible shape divergence along other intraspecific factors along the same axes of shape variance (RWs in Table 2), a feature inconsistent with the theoretical treatments above (but see [21]). This may be because the theoretical models described assume competition for resources generate the disruptive selection that drives trait divergence. It remains unclear, however, whether such a scenario applies to *T. cristinae*, as food resources seem readily available. Nevertheless, and regardless of the cause, mandible shape divergence between the sexes dominates that observed between host-adapted ecotypes and/or among populations. These findings contrast previous work in this species reporting strong divergence between host-adapted ecotypes in most other morphological features [4,25,27,29,38]. Thus, our data suggest that trophic morphology (as quantified here) does not strongly respond to the multifarious selection regime driving host-based ecotype adaptation, nor contribute to reproductive isolation between host-adapted ecotypes. It may instead limit this process by occurring along a very different intraspecific factor.

### Host and population based shape in wild males

Once the influence of sex was removed, mandible shape in wild males along a gene flow gradient was variably explained by different factors depending on the test performed. Discrepancies are likely the consequence of differential test sensitivities and the more subtle nature of within sex shape differences. MANOVA used all RWs weighted equally to establish groups differences, and may therefore be oversensitive to small differences in less important RWs. Likewise, Bayesian clustering also considered all RWs evenly (although see [76]) and found the most likely clustering based on normal or log-normal distributions with fixed error terms. If differences between groups are more subtle and organised in complex hierarchies, they may be more difficult to recover [46,76]. In contrast, the kernel density based MU weighs the contribution of each RW to group-specific differences in occupied morphospace, and may thus more accurately quantify morphological differences [49]. MU indices, however, cannot easily quantify interactions among factors and can therefore underestimate how they influence overall shape [48]. Nevertheless, and taking into account these discrepancies, lingual mandible shape variation was not consistent with host-based adaptive feeding. If it were, *A*-ecotypes would be expected to develop shapes that more easily initiate and/or propagate fractures in tougher more resistant materials [16,35,73]. Pointed sharp blades would do this best [16,73]. Contrary to these expectations, and although MU results showed some morphospace partitioning between host-adapted ecotypes, this was not well reflected in shape differences outlined by the first three RWs. Moreover, pairwise population level comparisons recovered as many significant MUs between populations using the same host as those using alternate ones. Thus lingual mandible shapes, chosen to reflect more functional features, were likely least influenced by host level adaptations. Variation in the occlusial mandible, however, showed significant levels of host-adapted ecotype variation. Separation in occupied morphospace by the different host-adapted ecotypes was clear along RW1, where *A*-ecotypes had shapes more appropriate for a harder, tougher host plant. Host-adapted ecotype divergence was less than that recovered in pairwise population comparisons, especially when only significant comparisons were considered. However, all significant population comparisons occurred between populations using alternate hosts suggesting some important host-population interaction not obvious in the lingual mandible. Thus, in the occlusial mandible more reflective of structural features, results suggest some influence of host plant adaptation, but only once the influence of other factors have been minimised (sex and rearing condition). These results suggest that host adaptation interacts extensively with population level differences and is specific to certain features accentuated in the different mandible orientations.

## Conclusions

Collectively, presented data show that mandible shape in *T. cristinae* is under both genetic and environmental control, mostly based on sexual differences, and to a lesser degree on differences between hosts and among populations. Mandible shape divergence in *T. cristinae* occurs predominantly along a different intraspecific factor than most other morphological traits and may, as a consequence, limit the progress toward ecological speciation between host-adapted ecotypes. Future work testing the causes of sexual dimorphism in trophic morphology in this species is required.

## Availability of supporting data

The landmark data for each individual is stored through Labarchives.com and is available at the following link: http://dx.doi.org/10.6070/H4GQ6VPM.

## Competing interests

The authors declare that they have no competing interests.

## Authors’ contributions

PN and DR conceived the study and design. DR formulated metrics, wrote appropriate scripts and conducted analyses. DR, OS and PN wrote the manuscript. All authors read and approved the final submission.

## Acknowledgements

Chris Robinson, Brian Sinnet and Christa Jolidon form the EAWAG Dübendorf assisted with ESEM protocols and laboratory preparation of samples. D. Mouillot provided base scripts and Tarn Duong assisted with use of ks packages in R. This work was funded by a European Research Council Starter Grant (NatHisGen R/129639) to PN.

## Supplementary Material

Additional file 1 **Supporting tests and comparisons.** Supporting online information for this study outlines both common and global morphospace analyses, population level MU comparisons and relationships assessed between shape variation and the potential for gene flow among populations. Click here for file
