# Supplementary material for: Sexual dimorphism dominates divergent host plant use in stick insect trophic morphology
Source: BMC Evol Biol. 2013 Jul 3;13:135. doi: 10.1186/1471-2148-13-135 (PMC3707739; doi:10.1186/1471-2148-13-135)
Supplement: Additional file 1 — Supporting tests and comparisons. Supporting online information for this study outlines both common and global morphospace analyses, population level MU comparisons and relationships assessed between shape variation and the potential for gene flow among populations. [file 1471-2148-13-135-S1.pdf]

Sexual dimorphism dominates divergent host plant use in stick insect trophic morphology

Supplementary Information: Denis Roy, Ole Seehausen and Patrik Nosil

Common Morphospace

Common morphospace comparisons were done using ordered axes analyses [1]. Briefly, ordered axes analyses rank RW scores along the most important RWs for both males and females. Ranked scores form ordered variables that are regressed onto one another in two-dimensional space. If the regression equation fits a 1:1 relationship going through the origin, shape changes in both groups follow the same shape change trajectory and both groups accumulate shape diversity at the same rate. Deviations of the regressions from an intercept of zero, and/or a slope of 1 indicate different shape change trajectories or the unequivocal accumulation of shape diversity in either group (depending on the magnitude of the slope). We tested male and female shape changes along the first four RWs, which accounted for 65.96% and 64.15% of the overall shape changes for the lingual and occlusial mandibles, respectively. The slope and intercept of each regression, was tested for departure from a 1:1 relationship using 10 000 randomizations and jack-knifing iterations performed in PopTools v3.2.5 [2]. Ordered axes results showed that although diversity in both mandibles tended to accumulate at slightly different rates in males and females (slopes different from one) both sexes followed the same shape change trajectories when considered in common morphospace (Figure S1). Thus, divergence along the most important RWs tend to follow the same general trends for both the lingual and occlusial mandibles in male and female *T. cristinae* sampled here.

Global Morphospace

Shape changes between sexes were also compared in global morphospace wherein the major axes of shape change defined for each sex were determined separately and tested for parallel divergence. Parallel divergence axes among groups in global morphospace imply that considered groups are not different from a random subset of either group and can thus be treated as a single unified group [1, 3, 4]. Size corrected shape variables for both males and females were entered into the program SpaceAngle6b where sex specific RWs were calculated [1, 4, 5]. The angle between sex specific axes of divergence in morphospace defined by RW1, RW1-RW2, RW1-RW3 and RW1-RW4 were calculated, along with their 95% confidence intervals, as described in [4] and tested for parallelism. Within sex distribution of angles were calculated by randomly resampling two groups from both male and female shape data 4900 times (with replacement; maximum allowable by the program) and calculating their 95th percentiles (see Table S1). We then tested whether the between sex distribution of angles was greater than the 95th percentiles of both within-sex angles [1, 4].

None of the between sex angles estimated using RW1, RW1 and 2, RW1-3 and RW1-4 were found to be larger than both 95th percentile angles determined from the within sex distributions. These data indicate that although both males and females may not necessarily occupy the same region of global morphospace, they nevertheless appear to have similar shape change trajectories within it.

Table S1. Comparisons of angles between axes of divergence in global morphospace between male and female *T. crisitinae* mandible shapes.

Dimension*	Between Sexes	Between Sexes 95% C.I.	Within Males 95th percentile	Within Females 95th percentile
Lingual				
RW1 90	34.58°	20.90° - 88.17°	22.26°	88.17°
RW1-2 127	31.51°	30.15° - 85.90°	87.94°	54.68°
RW1-3 156	78.57°	56.40° - 99.23°	83.30°	90.34°
RW1-4 180	83.30°	74.14° - 113.33°	80.20°	96.89°
Occlusial				
RW1 90	25.30°	21.13° - 47.71°	30.81°	51.01°
RW1-2 127	81.92°	45.60° - 92.27°	64.93°	87.94°
RW1-3 156	54.61°	48.80° - 95.40°	44.81°	89.52°
RW1-4 180	86.96°	69.90° - 106.45°	86.69°	93.46°

* Testing angles between 0 and upper bound defined by RWs considered.
Axes of divergence are considered significantly different if the distribution of between sex angles does not overlap with that between 0 and the sex-specific 95th percentile.

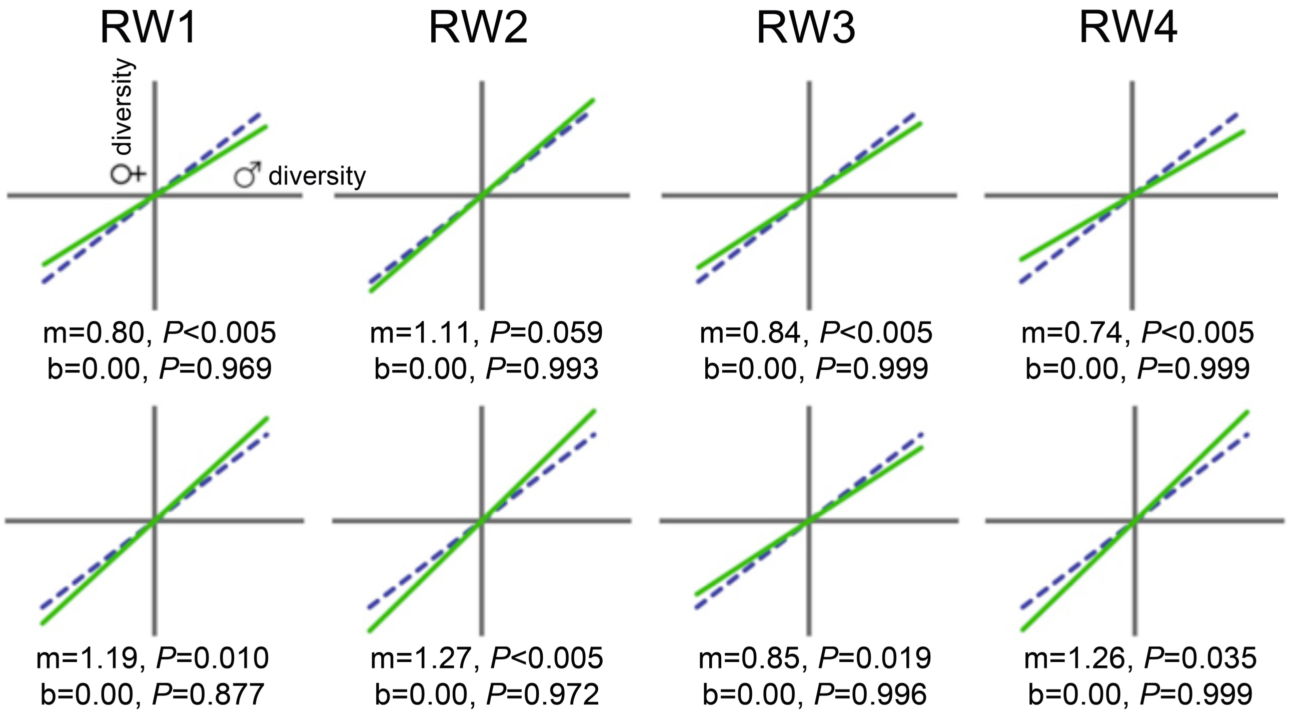

Figure S1. Ordered axes plots for both the lingual (top) and occlusial (bottom) mandibles of sampled *T. cristinae* along the first four RWs. Male diversity indicated along the x- and female diversity along the y-axes. Dashed lines represent the null model of similar shape change trajectory (intercept= 0) and the even accumulation of shape diversity in both sexes (slope = 1). Actual estimated relationship depicted by solid line and tested from deviation from the null using 10 000 jack-knifing permutations. m = slope and b = intercept of actual male-female relationships.

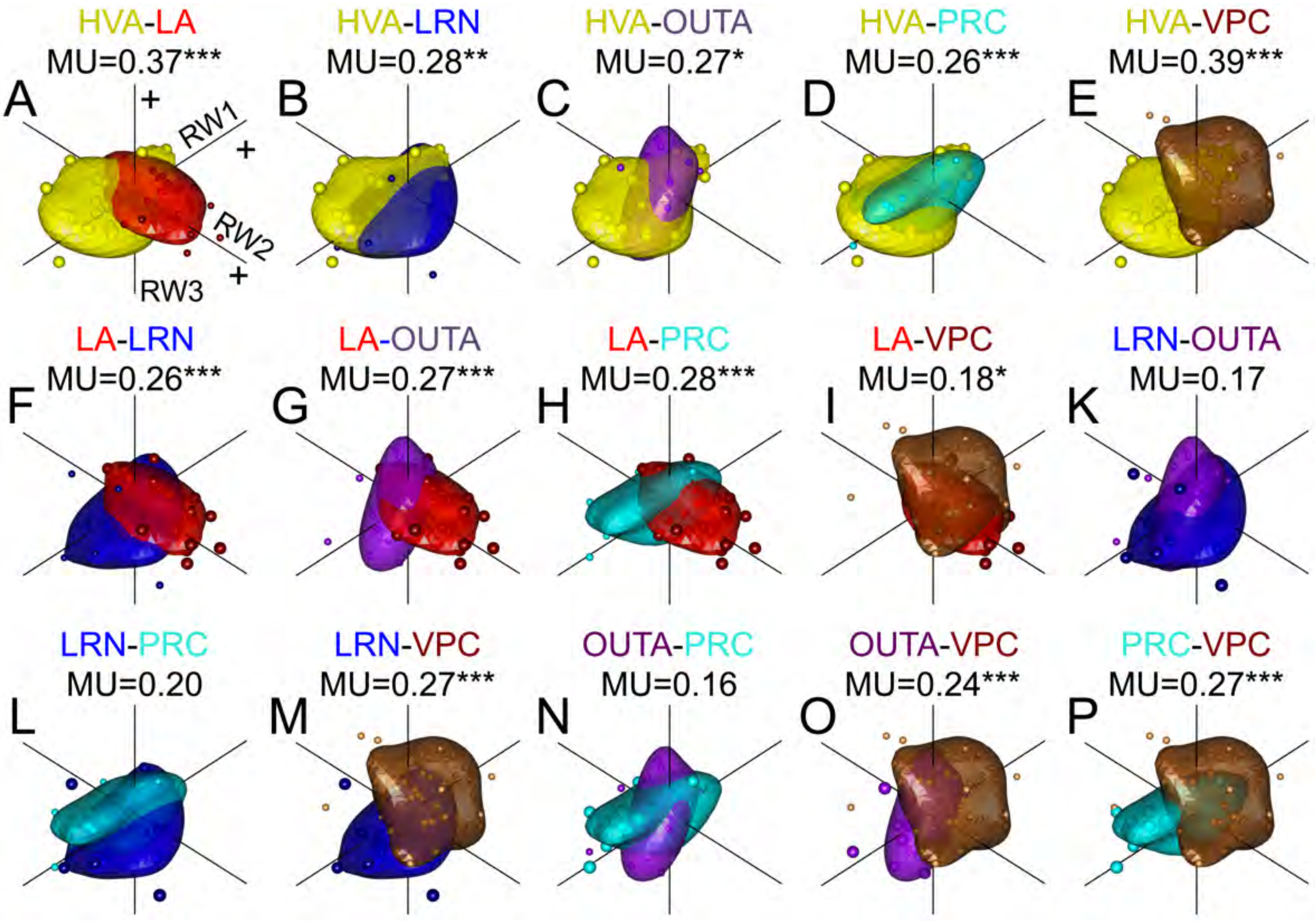

Figure S2. Pairwise Morphological uniqueness (MU) of lingual mandibles assessed between populations of *T. cristinae*. Estimates generated along all18 RWs supported by 1000 permutations shuffling the same individuals randomly in either population. Panels show 80% confidence bubbles outlining morphospace occupied by different populations along the first three RWs accounting for 57% of overall shape variation. Mean MU over all pairwise comparison = 0.26 (RW1=31.4%, RW2=14.6%, RW3=11.0%; *** = P<0.001, ** = P<0.01, and * = P<0.05).

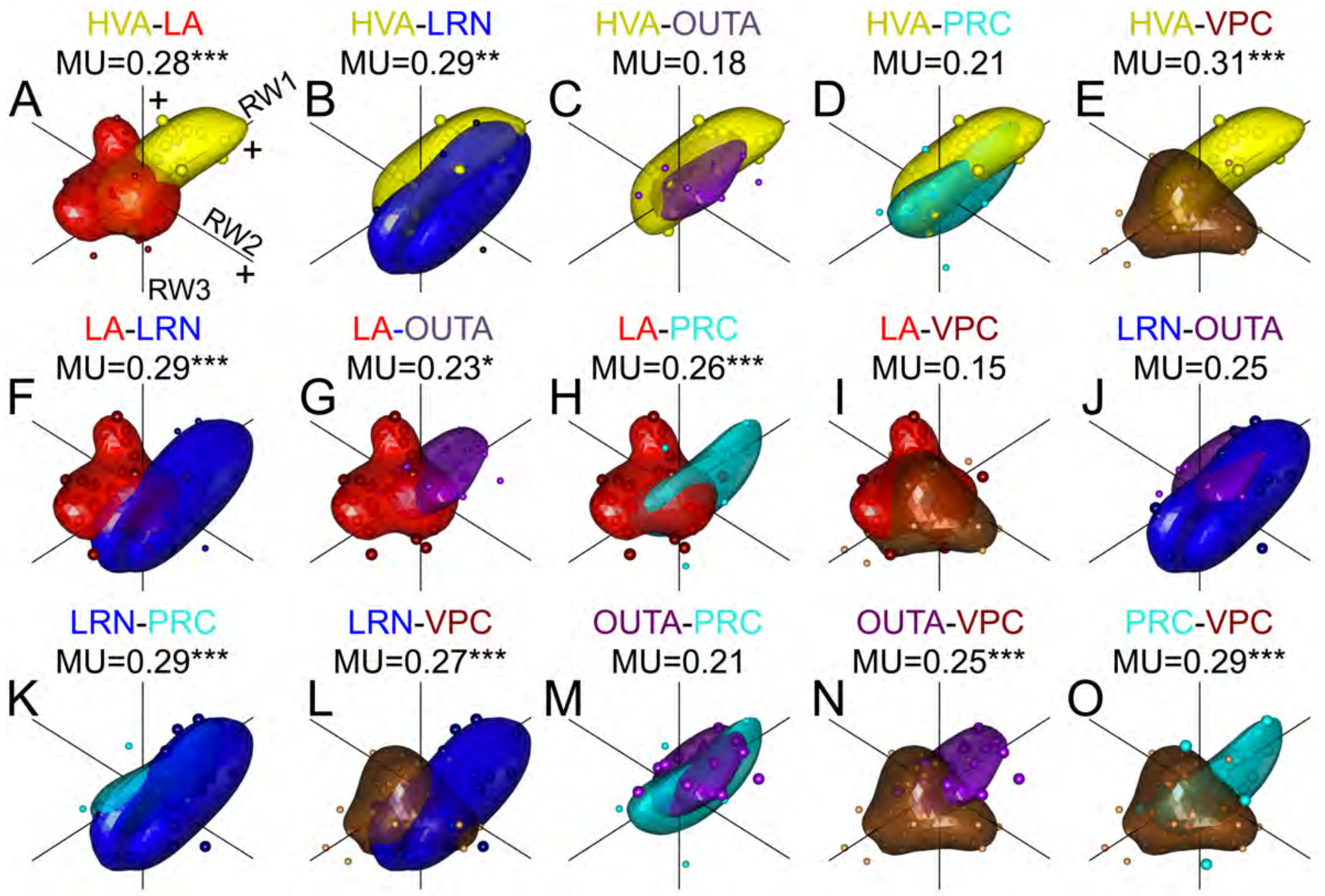

Figure S3. Pairwise Morphological uniqueness (MU) of occlusial mandibles assessed among populations of *T. cristinae*. Estimates generated along all18 RWs supported by 1000 permutations shuffling the same individuals randomly in either population. Panels show 80% confidence bubbles outlining morphospace occupied by different populations along the first three RWs accounting for 56% of overall shape variation. Mean MU over all pairwise comparisons = 0.25 (RW1=27.8%, RW2=14.3%, RW3=13.9%; *** = P<0.001, ** = P<0.01, and * = P<0.05).

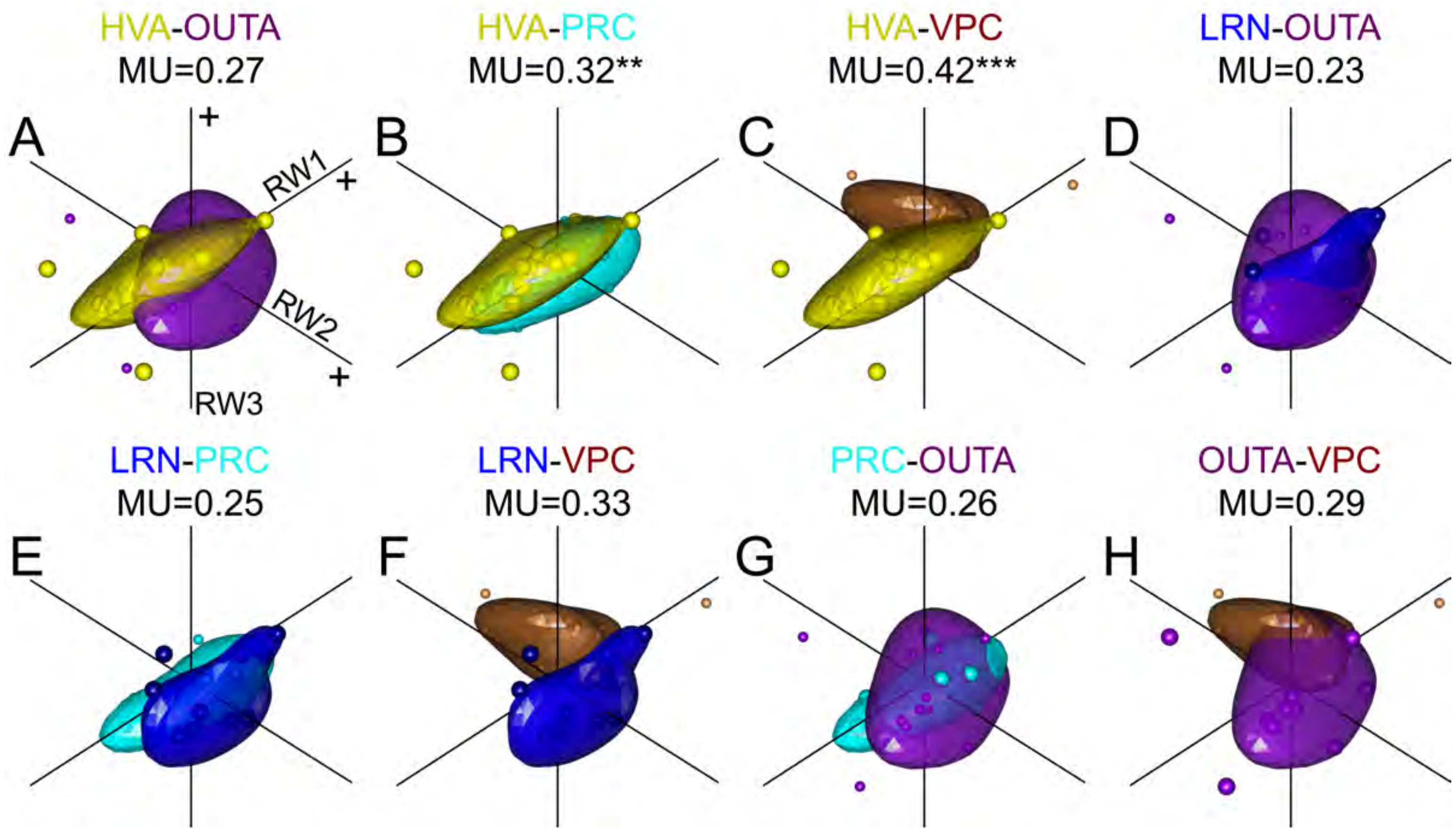

Figure S4. Pairwise population MU of lingual mandibles assessed using only field collected male *T. cristinae*. Estimates generated along all 18 RWs supported by 1000 permutations shuffling the same individuals randomly in either population. Panels show 80% confidence bubbles outlining morphospace occupied by the different populations along the first three RWs accounting for 60.3% of overall shape variation. Mean over all pairwise comparisons = 0.30 (RW1=32.6%, RW2=17.2%, RW3=10.5%; *** = P<0.001, ** = P<0.01, and * = P<0.05).

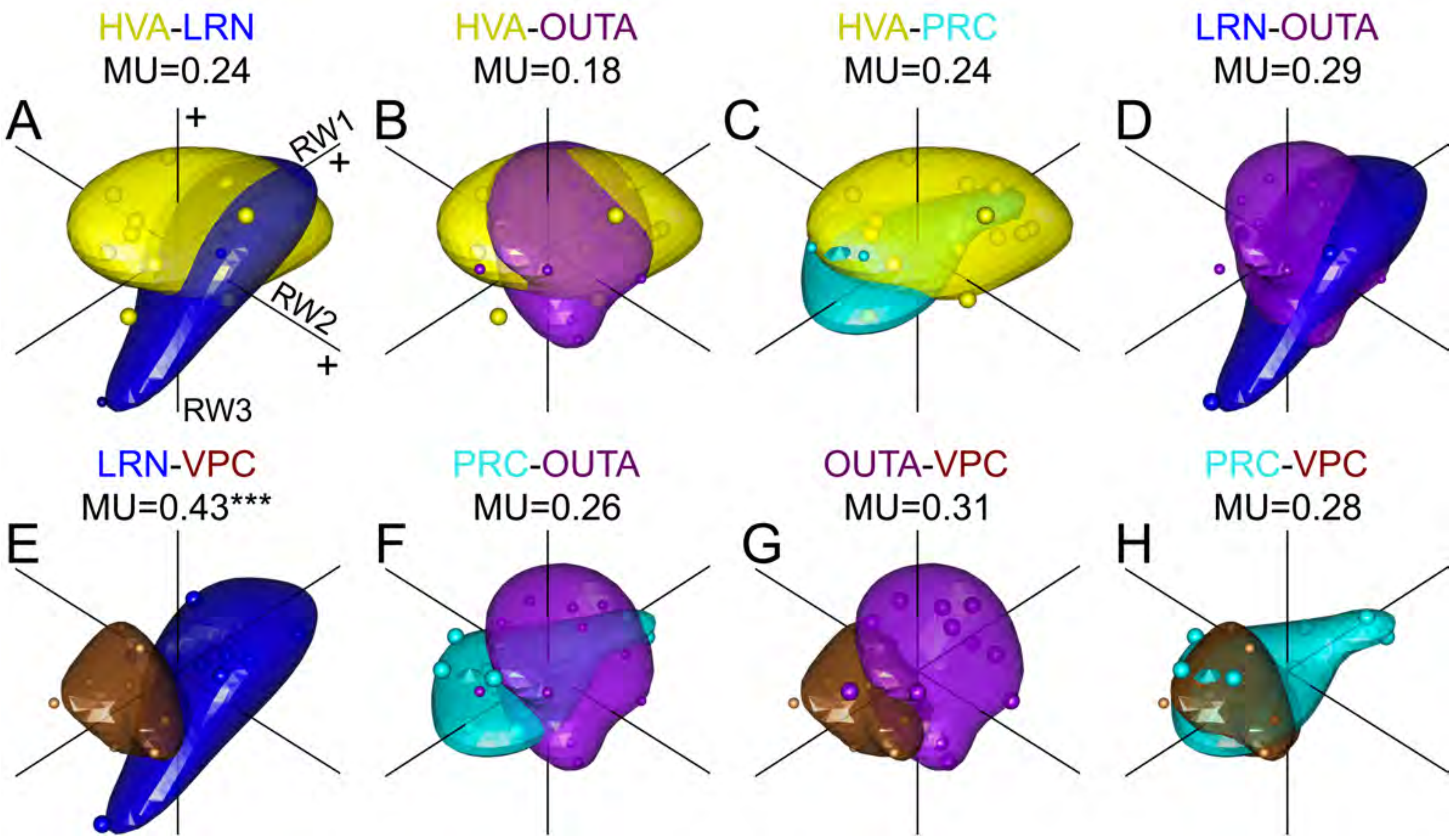

Figure S5. Pairwise population MU of occlusial mandibles assessed using only field collected male *T. cristinae*. Estimates generated along all 18 RWs supported by 1000 permutations shuffling the same individuals randomly in either population. Panels show 80% confidence bubbles outlining morphospace occupied by the different populations along the first three RWs accounting for 67% of overall shape variation. Mean MU over all pairwise comparisons = 0.29 (RW1=38.9%, RW2=16.4%, RW3=11.7%; *** = $P<0.001$, ** = $P<0.01$, and * = $P<0.05$).

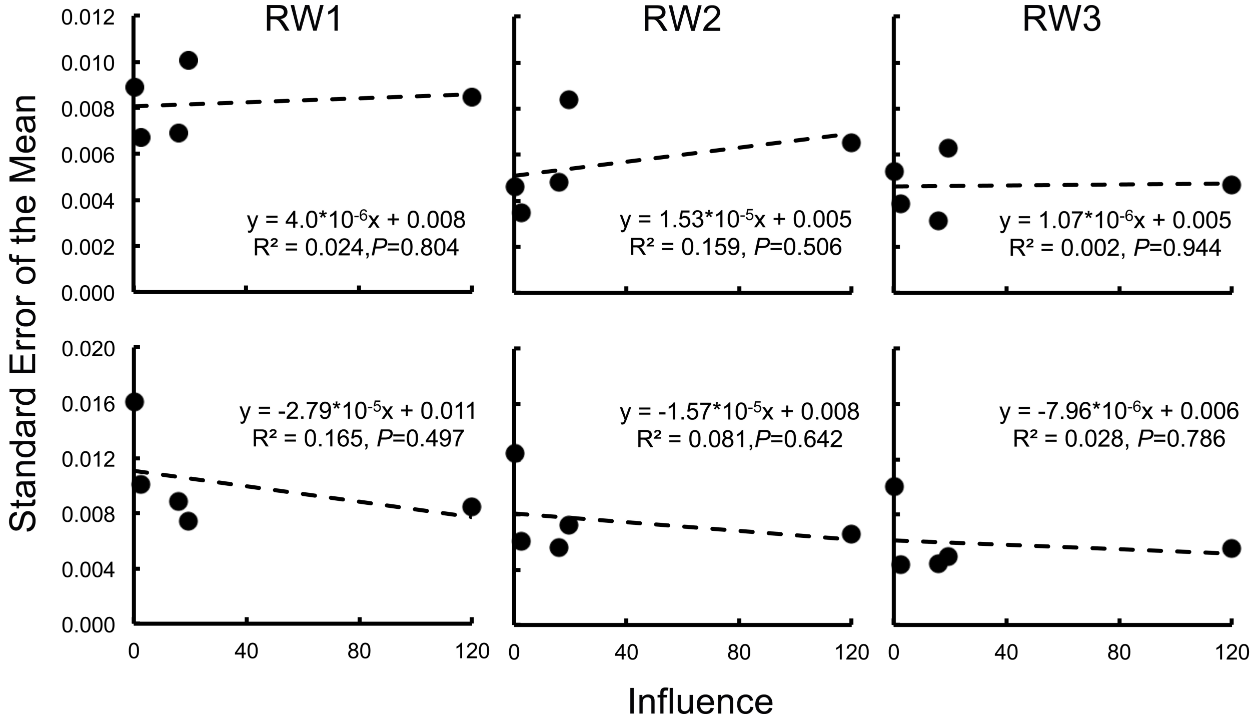

Figure S6. Gene flow effects on mandible shape variance. Potential gene flow between neighbouring populations (assessed as influence; see text) and the variance (expressed as standard error of the mean) along the three most important RWs for both the lingual (top) and occlusial (bottom) mandible shapes in field collected male *T. cristinae*. First three RW explain 60.3% and 67% of the overall shape variation in the lingual and occlusial mandibles, respectively.

References

1. Young KA, Snoeks J, Seehausen O: **Morphological diversity and the roles of contingency, chance and determinism in african cichlid radiations.** *PLoS ONE* 2009, **4**(3).
2. Hood GM: **PopTools**. Version 3.2.5. 2011. available on line at http://www.poptools.org
3. Mezey JG, Houle D: **Comparing G matrices: Are common principal components informative?** *Genetics* 2003, **165**(1):411-425.
4. Zelditch ML, Swiderski DL, Sheets HD, Fink WL: **Geometric Morphometrics for Biologists: A Primer.** Amsterdam, The Netherlands.: Elsevier, Academic Press.; 2004.
5. Sheets HD: **SpaceAngle**. 6b. 2007. available on line at http://www2.canisius.edu/sheets/morphsoft.html.
